# Flavivirus Entry Receptors: An Update

**DOI:** 10.3390/v6010069

**Published:** 2013-12-30

**Authors:** Manuel Perera-Lecoin, Laurent Meertens, Xavier Carnec, Ali Amara

**Affiliations:** 1 INSERM U944, CNRS 7212, Laboratoire de Pathologie et Virologie Moléculaire, Hôpital Saint-Louis, 1 Avenue Claude Vellefaux, Paris 75010, France; E-Mails: laurent.meertens@inserm.fr (L.M.); xavier.carnec@gmail.com (X.C.); ali.amara@inserm.fr (A.A.); 2 Institut Universitaire d'Hématologie, Hôpital Saint-Louis, 1 Avenue Claude Vellefaux, Paris 75010, France; 3 Université Paris Diderot, Sorbonne Paris Cité, Hôpital St. Louis, 1 Avenue Claude Vellefaux, Paris 75475, France

**Keywords:** flavivirus, West Nile virus, dengue virus, viral entry, C-type lectin receptor, phosphatidylserine receptors

## Abstract

Flaviviruses enter host cells by endocytosis initiated when the virus particles interact with cell surface receptors. The current model suggests that flaviviruses use at least two different sets of molecules for infectious entry: attachment factors that concentrate and/or recruit viruses on the cell surface and primary receptor(s) that bind to virions and direct them to the endocytic pathway. Here, we present the currently available knowledge regarding the flavivirus receptors described so far with specific attention to C-type lectin receptors and the phosphatidylserine receptors, T-cell immunoglobulin and mucin domain (TIM) and TYRO3, AXL and MER (TAM). Their role in flavivirus attachment and entry as well as their implication in the virus biology will be discussed in depth.

## 1. Introduction

Within the *Flaviviridae* family, the genus, *Flavivirus*, encompasses more than 70 enveloped viruses, many of which are transmitted to vertebrates by the bite of hematophagous arthropods, such as mosquitoes and ticks. These viruses are important human pathogens that represent an emerging public health problem, due to the rapid geographical spread of the vectors and the high morbidity and mortality of these infections [1]. Among flaviviruses, dengue virus (DENV) and yellow fever virus (YFV) are able to cause hemorrhagic fevers, while West Nile virus (WNV), tick-borne encephalitis virus (TBEV), Murray encephalitis virus (MEV) and Japanese encephalitis virus (JEV) are the causative agents of potentially lethal neurological diseases, such as encephalitis and meningitis [2]. 

Due to the rising incidence of flavivirus infection worldwide, increasing efforts have been made in the last decade to understand their biology. As a result, significant progress has been achieved in several fields. For instance, flavivirus research has enormously benefited from structural studies, in particular, of the envelope (E) glycoprotein and from cryo-electron microscopy reconstructions of intact virions, which have been turning points for understanding their organization. However, many aspects of the cell biology of flaviviruses, such as the molecular interactions they use to enter cells and the identity of the cellular receptors involved in virus binding and internalization, are far from being understood to the same extent. 

The entry of flaviviruses into their target cells is mediated by the interaction of the E glycoprotein with cell surface receptors. Receptor recognition and attachment is likely to be a process in which multiple molecules are used in combination or consecutively for infectious entry. Several studies have indicated that flaviviruses make initial contact with the host cell by binding to glycosaminoglycans (GAGs), such as heparan-sulfate proteoglycans or syndecans [3,4,5,6,7,8]. GAGs are long, unbranched, sulfated polysaccharides that are found linked to core proteins attached to cellular surfaces (proteoglycans) [9]. GAGs are prominently exposed on the cell surfaces of all tissues, providing an easily accessible receptor for viral adhesion. Flavivirus binding to GAGs involves primarily the electrostatic interaction of clusters of positively charged residues on the surface of the E glycoprotein with negatively charged sulfate groups on the polysaccharide [3]. GAGs act mainly as attachment factors that concentrate flavivirus particles at the target cell surface before their interaction with primary receptors [3,4,5,6,7]. Despite an intense investigation, the identity of the cellular receptors that mediate flavivirus entry and infection is, at present, poorly known. A large number of molecules have been described as flavivirus candidate receptors in different cell types, but their precise role in virus endocytosis remains obscure (Table 1). Among all of them, only the α_v_ß_3_ integrin has been shown to function as a primary receptor on mammalian cells for a lineage II WNV and for JEV [10]. However, recent studies proved that a lineage I WNV strain was capable of infecting and replicating in cells lacking α_v_ß_3_ integrin [11]. Certainly, these findings do not eliminate a role for α_v_ß_3_ integrin during the virus infection process, but they do suggest that α_v_ß_3_ integrin is not absolutely required for entry in every cellular context. Currently, the best-characterized protein families that bind to and enhance flavivirus infection are C-type (calcium-dependent) lectin receptors and the recently identified phosphatidylserine receptors, T-cell immunoglobulin and mucin domain (TIM) and TYRO3, AXL and MER (TAM), which will be discussed in detail in the following sections. 

## 2. C-Type Lectin Receptors

Cellular C-type lectin receptors (CLRs) are specialized in sensing invading pathogens. Several members of this family are highly expressed on myeloid cells, including monocytes, macrophages and dendritic cells (DCs), and thus, play a central role in activating host immune defenses. CLRs recognize carbohydrate profiles on pathogens and act as internalization receptors that target pathogens to acidified endosomes for antigen presentation and pathogen clearance (reviewed in [12]). Flaviviruses may have evolved to exploit multiple CLRs for viral dissemination.

**Table 1:** Properties and expression of the different flavivirus receptors proposed to date. TIM: T-cell immunoglobulin and mucin domain; TAM: TYRO3, AXL and MER; DENV: dengue virus; JEV: Japanese encephalitis virus; MEV: Murray encephalitis virus; TBEV: tick-borne encephalitis virus; YFV: yellow fever virus; WNV: West Nile virus.

Molecule	Properties	Cells	Virus	References
Heparan sulfate	Glycosaminoglycans	Mammalian	DENV, JEV, MEV, TBEV, YFV, WNV	[3,4,5,6,7,8]
GRP78/ HSP70/90	Heat-shock proteins	Mammalian, mosquito	DENV, JEV	[13,14,15,16,17,18,19,20]
DC-SIGN/L-SIGN	C-type lectins	Mammalian Mammalian Mammalian	DENV, WNV	[21,22,23,24,25,26]
Mannose receptor			DENV	[27]
CLEC5A			DENV, JEV	[28,29,30]
mosGCTL-1		Mosquito	WNV	[31]
Laminin receptor	High-affinity laminin receptor	Mammalian, mosquito	DENV, WNV	[32,33]
Prohibitin	Modulator of mitochondrial function and transcription	Mosquito	DENV	[34]
TIM receptors	Phosphatidylserine receptors	Mammalian	DENV, WNV, YFV	[35,36]
TAM receptors		Mammalian	DENV, WNV, YFV	[35]
Integrin α_v_β_3_	Vitronectin receptor	Mammalian	DENV, WNV, JEV	[10,37]
Scavenger receptor Class B type I	High-density lipoprotein receptor	Mammalian	DENV	[38]
Claudin-1	Tight junctions component	Mammalian	DENV	[39,40]
NKp44	Natural Killer cell activating receptor	Mammalian	DENV, WNV	[41]

### 2.1. Dendritic Cell-Specific Intercellular Adhesion Molecule-3-Grabbing Non-Integrin (DC-SIGN) and Liver/Lymph Node-Specific ICAM-3 Grabbing Non-Integrin (L-SIGN)

DC-SIGN and L-SIGN are type 2 transmembrane C-type lectins. Their extracellular domains share common structural motifs, including an extended neck composed of tandem repeats of a highly conserved 23-amino acid sequence, followed by a carbohydrate recognition domain (CRD), which binds mannose-rich glycans [42]. DC-SIGN is highly expressed on some macrophage subsets and immature DCs [43,44], which are thought to facilitate viral dissemination [45,46]. L-SIGN expression is restricted to sinusoidal endothelial cells in the liver and endothelial cells in the lymph nodes [47,48,49]. Despite evidences that L-SIGN-expressing cells are infected *in vivo*, the role of L-SIGN during the course of natural infection has not been clearly established yet [50,51]. The importance of the interaction with DC-SIGN in flavivirus tropism and pathogenesis is exemplified by the recent identification of a single nucleotide polymorphism in the DC-SIGN promoter that is associated with a predisposition to severe forms of dengue hemorrhagic fever (DHF) and tick-borne encephalitis [52,53].

DC-SIGN and L-SIGN are able to bind and promote infection of both DENV and WNV by interacting with *N*-linked glycans on E protein [20,21,22,23,24,25]. Despite the structural similarity between the two viruses, DENV efficiently infects DC-SIGN- and L-SIGN-expressing cells, while L-SIGN promotes WNV infection more efficiently than DC-SIGN, at least when the virus is grown in mammalian cells. However, both DC-SIGN and L-SIGN can promote infection to the same extent when WNV is grown in mosquito cells [20,24,25]. The major structural differences between DENV and WNV virions that account for this discrepancy are the number of *N*-linked glycosylation sites and their location on the E protein, as well as the type of glycans associated with these sites.

Most DENV E proteins have two potential glycosylation sites, at residues Asn67 and Asn153. Asn67 is unique for DENV, whereas Asn153 (and the corresponding Asn154 in WNV) is conserved among flaviviruses [54]. Structural studies have shown that binding of DENV to DC-SIGN involves the preferential interaction of the CRD with the glycosylated Asn67 on the E protein [55]. This amino acid is critical for DC-SIGN-dependent infection, since DENV molecular clones lacking this glycosylation site do not infect DC-SIGN-expressing cells. Consistently, introducing Asn67 into WNV E protein enhances the infection of DC-SIGN-positive cells [23,24]. This is likely due to Asn67 spatial arrangement, which fits more than Asn153 with the CRD of tetrameric DC-SIGN [55]. 

Another crucial factor is the type of *N*-linked glycans attached to E protein glycosylation sites. Several reports have shown that viruses grown in mosquito or mammalian cells display different *N*-linked glycans, and this correlates with the usage of DC-SIGN and L-SIGN [24,25,26,27,28,29,30,31,32,33,34,35,36,37,38,39,40,41,42,43,44,45,46,47,48,49,50,51,52,53,54,55,56]. Mosquito cells have a limited capacity to process oligosaccharides, which conserve a terminal mannose residue. Therefore, the E protein of virions grown in insect cells displays high-mannose glycans that are recognized by both DC-SIGN and L-SIGN [20,24]. In contrast, mammalian cells are able to produce complex glycans with residues, such as N-acetylglucosamine, that are preferentially recognized by L-SIGN [20,24,25]. These observations may explain why WNV grown in mosquito cells (and probably other flaviviruses) can bind efficiently and infect DC-SIGN-expressing cells in contrast to virus grown in mammalian cells.

Interestingly, DENV grown in mammalian cells retains the ability to bind to and infect DC-SIGN-expressing cells. It has been proposed that the proximal mannose residue of Asn67 *N*-linked glycan may be covered by the viral prM protein and thus, cannot undergo complete processing by Golgi mannosidase [24]. Indeed, prM associates with the E protein during assembly and transit through the trans-Golgi network, and this could hide mannose residues from enzymatic activity. This hypothesis is consistent with the demonstration that DENV virions grown in mammalian cells retain sensitivity to EndoH digestion, indicating the presence of at least one high-mannose glycan [25,56].

**Figure 1:**
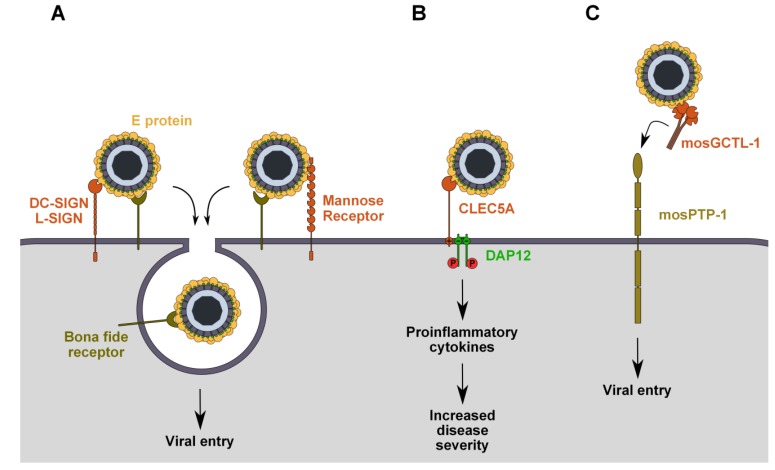
Attachment of flaviviruses to C-type lectin receptors has different implications in flaviviruses infections. (**A**) In mammalian cells, DC-SIGN/L-SIGN and the mannose receptor (MR) act as attachment factors that bind virions and facilitate their entry by transferring them to the *bona fide* receptor(s) involved in endocytosis; (**B**) In mammalian cells, binding of flaviviruses to CLEC5A triggers DAP12 phosphorylation and downstream signaling pathways that lead to the release of pro-inflammatory cytokines and aggravation of the disease; (**C**) In mosquitoes, virus entry is facilitated by the binding of virus/mosGCTL-1 complexes to cellular mosPTP-1.

DC-SIGN and L-SIGN share conserved internalization motifs in their cytoplasmic tails that are essential for endocytosis. These are a di-leucine motif and a cluster of three acidic amino acids that are responsible for endocytosis and targeting to late endosomes and lysosomes, respectively [57,58]. Therefore, these molecules could be good candidates as *bona fide* flavivirus receptors, allowing virus binding and internalization and ensuring the delivery to acidic endosomes, where membrane fusion will occur. However, mutation of the internalization motifs or deletion of the entire cytoplasmic tail, which inhibits antibody-induced DC-SIGN internalization, does not abolish DENV entry. This highlights the docking function of DC-SIGN, anchoring virions that are then delivered to a secondary molecule (or set of molecules) responsible for virus internalization (Figure 1A) [59].

### 2.2. Mannose Receptor

The mannose receptor (MR) is another CLR that has been proposed as a functional receptor of DENV [26]. Unlike DC-SIGN and L-SIGN, MR has multiple CRD-like domains and a cysteine-rich domain (CR) at the extremity of its extracellular domain that is able to interact with sulfated sugars [41]. The MR is essentially expressed on macrophages, but it can also be found on lymph nodes and liver endothelial cells, on kidney cells and on some DC populations, all of which are relevant to flavivirus infection [60]. The MR recognizes different types of sugars and has been implicated in the clearance of endogenous glycoproteins, as well as in the uptake and processing of foreign mannosylated antigens in antigen-presenting cells (APCs) [61,62]. It is constitutively internalized from the plasma membrane by clathrin-dependent endocytosis, which is mediated by a tyrosine residue within a di-aromatic motif of its cytoplasmic tail [63]. The MR has been shown to bind the E protein of all four DENV serotypes and has been proposed to be an internalization receptor for DENV in human primary macrophages, since polyclonal antibodies against the MR inhibit infection (Figure 1A) [26]. However, these data are insufficient to conclude that the MR is directly involved in DENV internalization. It is still unknown whether MR expression renders cells permissive to DENV and other flavivirus infection, as demonstrated with DC-SIGN. Furthermore, there is no *in vivo* evidence, such as human genetic polymorphism associations, implicating the MR in flavivirus pathogenesis. 

### 2.3. CLEC5A

C-type lectin domain family 5 member A (CLEC5A), which is expressed on monocytes and macrophages, is a CLR that has been shown to interact with DENV and JEV, although it is not yet clear if this binding involves interactions with glycans of the E protein. Unlike DC-SIGN and L-SIGN, it lacks a cytoplasmic tail with internalization motifs [64]. Instead, through a positively charged amino acid of its transmembrane domain, it associates with DNAX-activating protein 12 kDa (DAP12), an immunoreceptor tyrosine-based activation motif (ITAM)-bearing adapter molecule that transduces intracellular signaling [65]. JEV or DENV interaction with CLEC5A does not promote infection, but triggers the release of pro-inflammatory cytokines from macrophages and microglia through DAP12 phosphorylation, causing inflammation, vascular leakage and cell death, all of which have a lethal effect in mice (Figure 1B) [27,28,29]. These effects can be prevented by blocking virus-receptor interactions with neutralizing antibodies against CLEC5A, suggesting that this molecule plays a crucial role in flavivirus pathogenesis, particularly in the progression toward the severe forms of disease. The role of CLEC5A in WNV infection remains to be investigated. 

### 2.4. mosPTP-1/mosGCTL-1

Based on RNAi screening that characterized several human proteins facilitating WNV infection [66], Cheng *et al*. identified their genetic homologs in mosquitoes and analyzed their expression in WNV-infected *Aedes aegypti*. Among these, genes with altered expression were further examined for the effect of their knockdown on virus infection. This study identified mosGCTL-1 (mosquito galactose-specific binding C-type lectin) as a critical host factor for WNV infection [30]. Indeed, in *Culex* and *Aedes* mosquitoes, secreted mosGCTL-1 enhances WNV infection by interacting with the virus and bridging it to the cellular receptor, mosPTP-1, a protein tyrosine phosphatase expressed at the cell surface (Figure 1C) [30]. This is particularly relevant from a physiological point of view, as *Aedes* and *Culex* mosquitoes are major WNV vectors. However, mosPTP-1 participation in virus endocytosis was not studied, and it is not clear if it acts as an attachment factor or as an entry receptor.

Little is known about the biological functions of mosGCTL-1/mosPTP-1 interactions. Both have human homologs (MBL and CD45, respectively) that can interact and regulate the development of thymocytes [67]. It would be interesting to study if these molecules can mediate the cell entry of flaviviruses in human cells, knowing that CD45 is expressed on hematopoietic cells that are essential for virus replication and dissemination [68,69].

## 3. Discovery of the Function of TIM and TAM during Flavivirus Entry

In an effort to identify new flavivirus entry factors, we recently carried out a gain of function cDNA screen for human genes that render poorly permissive 293T cells susceptible to DENV infection [34]. To our knowledge, this was the first time that such a strategy was adopted for flaviviruses. Most of the candidate receptors proposed so far have been characterized by affinity chromatography and virus overlay protein binding assay (VOPBA) techniques [12,13,14,15,16,17,18,19,31,32,33,36]. Some of them have been proposed to bind the virus and mediate infection, but only in restricted cellular models and by certain types of viruses [10,11,17,31,32]. Furthermore, there is no clear correlation between the expression of these molecules and flavivirus tropism, and data regarding their direct involvement in the endocytosis of virus particles are still missing. Therefore, to date, none of these molecules can be considered as *bona fide* receptor(s).

Our screen identified the T-cell immunoglobulin and mucin domain (TIM) members, TIM-1, TIM-3 and TIM-4, and TAM receptors, TYRO3 and AXL, as DENV and WNV entry factors [34]. These molecules belong to two distinct families of transmembrane receptors that bind to phosphatidylserine (PtdSer) [70,71], an “eat me” signal that promotes the engulfment of apoptotic cells [72,73]. 

### 3.1. TIM Receptors Directly Bind to Flaviviruses

In human, TIM-1, TIM-3 and TIM-4 constitute the TIM family. TIM-3 is essentially expressed by Th-1 cells [74] and TIM-1 by Th-2 cells [75], but also by epithelial cells [76], which are known to be relevant to flavivirus infection [77,78]. TIM-4, on the other hand, is exclusively expressed by antigen presenting cells, such as macrophages and DCs [79], which represent primary targets of mosquito-derived flaviviruses. The role of TIMs is not limited to the phagocytosis of apoptotic cells, since they also regulate innate and adaptive immunity (reviewed in [71]). TIMs are type I cell surface glycoproteins with common structural properties, including an extracellular region composed of an *N*-terminal immunoglobulin-like domain (IgV domain) and an *O*- and *N*-linked glycosylated mucin domain, a single transmembrane segment and a cytoplasmic tail with tyrosine phosphorylation motifs, with the exception of TIM-4. TIMs interact with PtdSer residues on apoptotic bodies through a conserved pocket in the IgV domain, termed the metal ion-dependent ligand-binding site (MILIBS), which is the molecular signature of the TIM receptor family [71,80].

We have shown that TIM-1 and TIM-4 ectopic expression massively enhances infection by all DENV serotypes and related flaviviruses, such as WNV and YFV [34]. Assessment of receptor expression has established a strong correlation between TIM-1 endogenous expression and permissivity to DENV infection in human cell lines. The importance of TIM-1 in flavivirus entry is also highlighted by the finding that DENV and WNV infection of permissive cells is inhibited by anti-TIM-1 antibodies or by silencing expression of this receptor with RNA interference [34]. 

**Figure 2:**
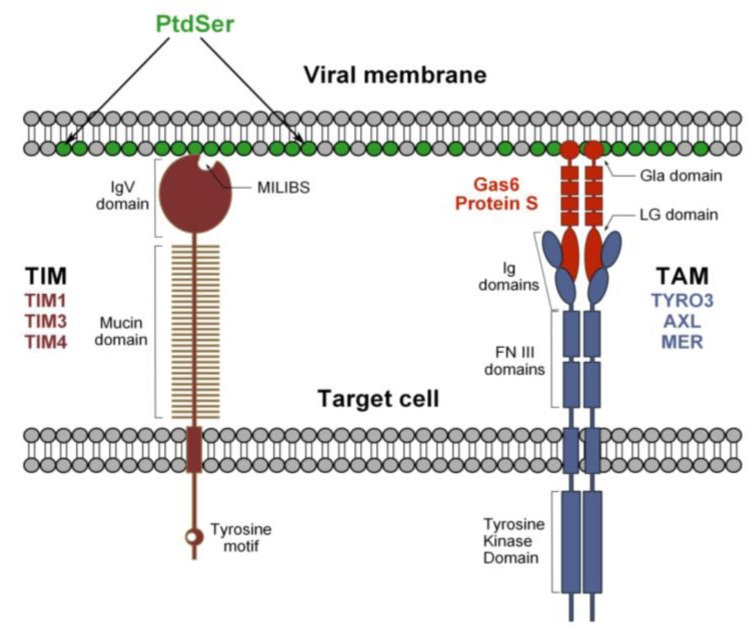
Hypothetical model for flavivirus recognition by TIM and TAM receptors. Phosphatidylserine (PtdSer) is expressed at the viral membrane and its recognition by TIM and TAM receptors occurs through a bimodal mechanism. The MILIBS pocket within the IgV domain of TIM receptors directly interacts with PtdSer. In contrast, the recognition of viral particles by TAM receptors is indirect and requires the presence of a TAM ligand, Gas6 or ProS. These molecules recognize both the virus-associated PtdSer via their Gla domain, and the TAM receptors through their LG domains and, thus, act as bridging factors.

TIMs interact directly with flavivirus particles and mutations of highly conserved amino acids (TIM-1 N114A or D115A, TIM-4 N121A) lining the MILIBS abolish infection. Moreover, annexin V, which specifically binds PtdSer, inhibits TIM-mediated enhancement of infection. Despite a lack of structural evidence, these findings strongly suggest that the interaction of TIMs with flaviviruses relies on the direct recognition of virion-associated PtdSer through the MILIBS pocket rather than the E protein (Figure 2) [34]. In agreement with this hypothesis, recent publications have shown that TIM-1 is able to enhance the transduction of different pseudovirions devoid of envelope glycoproteins or lacking intact receptor binding domains [35,81].

### 3.2. Tripartite Model for TAM Receptor Action during Flavivirus Entry

TYRO3, AXL and MER (TAM) receptors are protein tyrosine kinases (PTK) that regulate several immune responses [82], in particular the clearance of apoptotic cells and the inhibition of innate immunity [83,84]. TYRO3 is essentially found in the central nervous system, while MER and AXL are broadly expressed and can be found on APCs, such as monocytes and macrophages [85]. TAM receptors are single-path transmembrane proteins that can homodimerize and heteroligomerize. Their extracellular domain is composed of a tandem of two immunoglobulin-like domains (Ig) involved in ligand-binding and two fibronectin III (FNIII) repeats. The transmembrane domain is followed by the cytoplasmic tail, wherein lies PTK activity [86]. Growth-arrest-specific 6 (Gas6) and protein S (ProS), the two natural ligands of TAM receptors, have a similar domain organization: an *N*-terminal region containing 11 g-carboxyglutaminic acid residues (Gla), followed by four EGF-like domains, and a *C*-terminal region composed of two globular laminin G-like (LG) domains [83,87]. Contrary to TIM receptors, TAM receptors do not bind directly to apoptotic cells, but indirectly, through a process that requires the presence of Gas6/ProS, which function as bridging molecules. Their γ-carboxylated Gla domain interacts with PtdSer, while the LG domains bind to the Ig-like domains of TAM receptors [88,89]. DENV and WNV binding to TAM receptors occurs only in the presence of TAM ligands Gas6/ProS, which is akin to the mechanism of the recognition of apoptotic bodies (Figure 2). Indeed, we have also shown that mutations of conserved amino acids in the Ig-like domain involved in ligand binding abolish infection promoted by TAM receptor expression. The same has been noted with Gla-domain-deleted Gas6 [34]. These data argue for a tripartite model, whereby TAM ligands bind to PtdSer associated with DENV particles and bridge virions to TAM receptors. TYRO3 and AXL ectopic expression enhances infection by all DENV serotypes, as well as by the related WNV and YFV. Furthermore, infection of primary human cells or cell lines that are susceptible to flaviviruses, such as astrocytes or epithelial cell lines, relies on AXL endogenous expression, because antibodies against this receptor strongly block DENV infection [34]. 

### 3.3. Mechanism of TIM- and TAM-Mediated Enhancement of Flavivirus Infection

We have shown that binding of flavivirus particles to TIM-1 and AXL triggers virus uptake. However, the exact mechanisms involved remain to be investigated. TIM-1 could mediate flavivirus endocytosis, as it is constitutively internalized through clathrin-dependent endocytosis, a process that appears to rely on phosphorylation of tyrosine residues within the cytoplasmic tail [90]. These phosphorylation motifs are also involved in the activation of T-lymphocytes through the PI3K/Akt pathway [91,92], which is known to be activated by flaviviruses to prevent cell death [93]. Therefore, TIM-1-dependent signaling could have an effect on flavivirus entry and replication. Nonetheless, studies have shown that the ectopic expression of wild-type TIM-1 enhances the binding of apoptotic cells, but not necessarily their engulfment, suggesting that this receptor only acts as a tethering molecule [90]. Similar results have been obtained in TIM-4-dependent phagocytosis experiments, showing that the cytoplasmic and transmembrane domains of the receptor are dispensable for the engulfment of apoptotic cells. This process requires cooperation with other molecules, TIM-4 acting as a tethering molecule and its partners as “tickling” receptors that internalize bound particles [94]. Further assessment is needed to clarify whether flaviviruses exploit the TIM receptors’ ability to internalize and transmit signals for their productive infection, or if they use them as attachment factors that concentrate viral particles on the cell surface and facilitate their interaction with unknown endocytic receptor(s).

**Figure 3:**
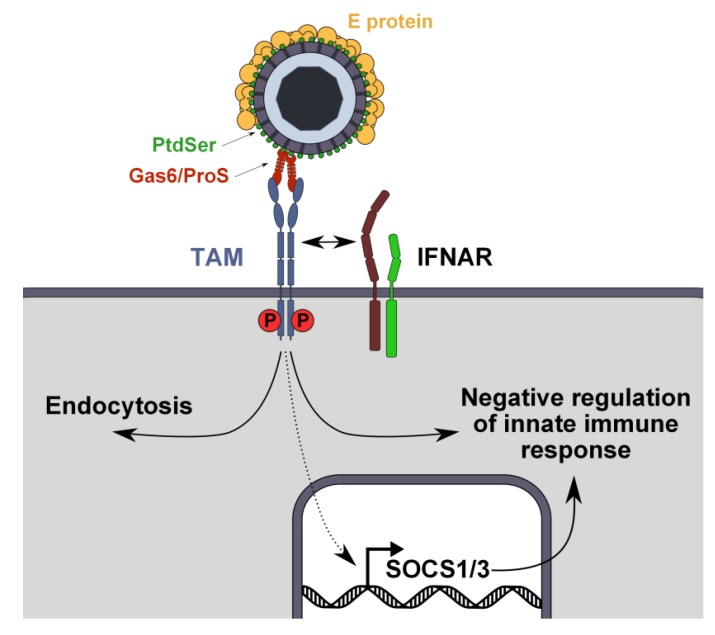
Dual role of TAM receptors during flavivirus infection. During entry, TAM receptors capture virus-Gas6/ProS complexes and enhance virus internalization through still unknown mechanisms. In parallel, virus-Gas6/ProS complexes activate TAM receptors, which recruit interferon receptor (IFNAR) to induce SOCS1/3 expression, thereby inhibiting innate antiviral responses and facilitating flavivirus replication.

In the case of TAM receptors, AXL could be also involved in the internalization of flaviviruses, as it has been implicated in the phagocytosis of apoptotic bodies and the macropinocytosis of Ebola virus, both known to rely on the PTK activity of its cytoplasmic domain [83,95,96]. However, we have shown that the deletion of the cytoplasmic tail of AXL or the mutation of the ATP-binding site essential for its kinase activity inhibit DENV infection, but not virus endocytosis [34]. This observation argues for a dual role of AXL during flavivirus infection: it can act as an attachment factor that transfers virions to an endocytic receptor in *cis*, but it may also act as a signaling molecule that regulates a post-entry step during the flavivirus lifecycle (Figure 3).

Interestingly, AXL kinase activity appears to be important for Ebola and Lassa virus infection [97,98]. TAM receptors kinase activity triggers signaling pathways involved in the regulation of survival through the PI3K/Akt pathway [99] and in the phagocytosis of apoptotic cells through the FAK/Ras module [100]. Moreover, upon Gas6 binding, AXL heterodimerizes with the type I interferon receptor (IFNAR) and enhances the transcription of suppressor of cytokines signaling genes *SOCS1* and *SOCS3*, which contribute to the pleiotropic inhibition of inflammatory cytokine and TLR signaling pathways [84]. One may speculate that Gas6 or ProS complexed to flaviviruses may act as “super AXL agonists” that modulate the host’s immune response to facilitate virus replication (Figure 3). Recent data support this concept, as WNV bound to TAM ligands downregulates type I IFN response by activating TAM receptors on murine DCs [101].

### 3.4. PtdSer Association to Flavivirus Envelope and TIM and TAM Accessibility

Our work has highlighted a crucial role of PtdSer in TIM- and TAM-mediated enhancement of flavivirus infection, which strongly suggests that this lipid is associated with the surface of the viral particles [34]. This raises important questions about how flaviviruses may incorporate PtdSer in their membrane and how this lipid becomes accessible to TIM and TAM receptors. The flavivirus membrane derives from the infected cell endoplasmic reticulum (ER) membrane, which is enriched in PtdSer in the luminal leaflet [102]. This suggests that flaviviruses incorporate PtdSer in their envelope when budding into the ER lumen. Further studies are required to confirm this hypothesis and to evaluate the involvement of other negatively charged lipids in the entry of flaviviruses, knowing that PtdSer receptors often recognize other anionic phospholipids, such as phosphatidylethanolamine [103]. 

**Figure 4:**
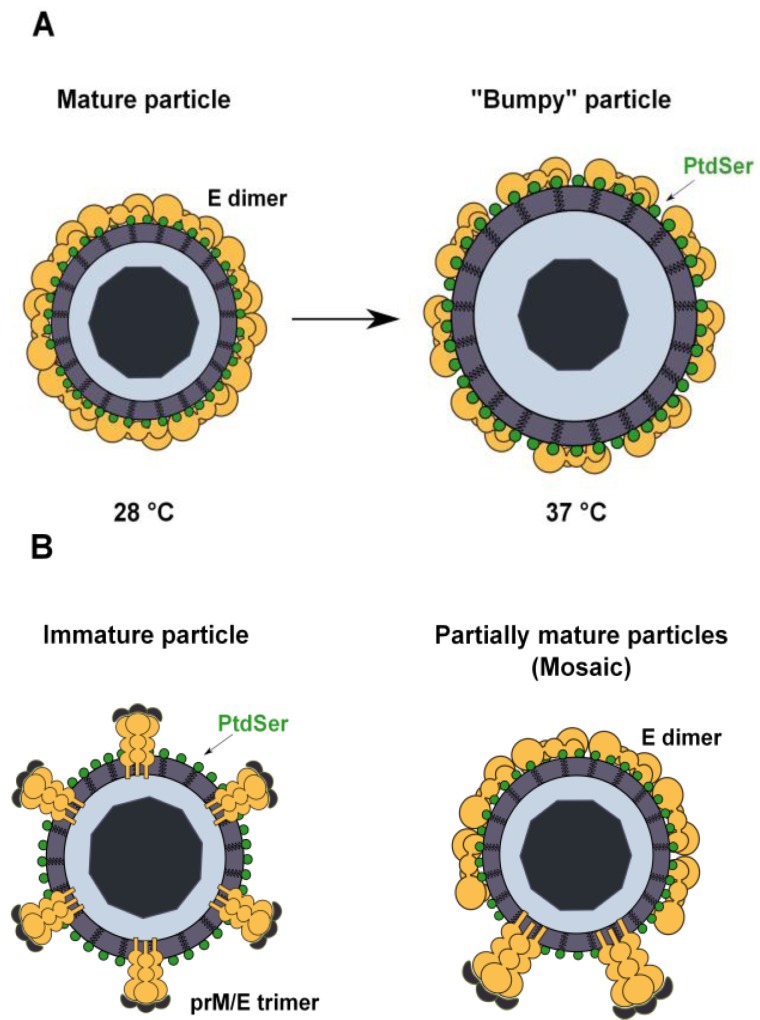
Possible mechanisms of PtdSer exposure in flavivirus virions. (**A**) Flavivirus particles produced in mosquito cells at 28 °C have a closed herringbone smooth conformation that protects the lipid envelope from the external medium. Upon an increase in temperature, particles expand and adopt a “bumpy” conformation that renders virion-associated PtdSer accessible. At 37 °C, the human body temperature, almost all virions present this conformation; (**B**) Inefficient cleavage of prM by cellular furin leads to the release of immature or partially mature (mosaic) virions in wich the lipid envelope is exposed to the external medium. Virion-associated PtdSer could therefore be accessible to TIM and TAM receptors.

A second major question is how TIM and TAM receptors access virion-associated PtdSer. Structural analyses show that mature flavivirus virions are composed of a nucleocapsid surrounded by the host cell membrane-derived lipid bilayer and a smooth spikeless outer glycoprotein shell. This closed smooth surface morphology is not compatible with the idea that PtdSer is accessible for TIM and TAM receptor usage. However, recent studies have shown that the DENV structure is dynamic and can be altered by temperature changes that can be encountered naturally when the virus passes from a mosquito to a human host [104,105]. Indeed, DENV virions produced in mosquito cells at 28 °C and exposed to temperatures up to 34 °C maintain the classical smooth herringbone conformation of the mature particles [105]. At higher temperatures, virions expand and adopt a “bumpy” conformation that could expose patches of the viral membrane, thereby allowing access of the TIM and TAM receptors to the virus membrane (Figure 4A) [104,105]. However, WNV virions produced at 37 °C retain a closed smooth conformation without exposing the virus membrane [106], which is not consistent with our data showing that TIM and TAM mediate the enhancement of WNV infection. 

Another explanation could be found in the inefficient cleavage of the prM protein during viral particle maturation. When virions acquire their lipid envelope during the budding of the nucleocapsid into the lumen of the ER, the resulting immature particles have a spiky surface, where E and prM are arranged as 60 heterotrimers [107,108]. The prM protein is proteolytically processed by cellular furin during transit through the trans-Golgi network, resulting in the production of mature virions [109,110]. However, this is an inefficient process, and some amount of uncleaved prM remains associated with virions. This leads to the release of a heterogeneous population of immature, partially mature and completely mature progeny virions [111,112]. The structural analysis of immature particles reveals a spiky surface composed of prM and E heterotrimers, while partially mature virion surfaces are mosaics with both spiky regions and smooth regions similar to those found in mature viruses [113,114,115]. Thus, the viral envelope can be exposed in these particles, which may greatly contribute to TIM and TAM receptor interaction (Figure 4B). Increasing evidences show that these virions remain infectious: they still bind to target cells through the interaction of prM with cellular lectins, and antibodies against prM can promote antibody-dependent enhancement of flavivirus infection [20,51,114,116,117,118]. 

## 4. Conclusions

The search for cellular receptors that mediate flavivirus entry is an active area of investigation. Functional studies have revealed the importance of CLR in the flavivirus lifecycle. However, there is still no clear evidence implicating these molecules in the direct internalization of virus particles. Thus, it is likely that these molecules act as attachment factors that concentrate virions at the cell surface and allow the possible interaction with endocytic receptors. We recently provided new insights into the cell biology of flavivirus infection and identified an unexpected role of PtdSer and the TIM and TAM proteins in the flavivirus entry program. This study suggests that, by mimicking apoptotic cells, flaviviruses manipulate the physiological functions of the TIM and TAM molecules for infection and probably to broaden their tropism. However, the role played by TIM and TAM receptors during flavivirus infection and pathogenesis is currently unknown, and many questions remain to be answered. First, further cell biology and virological studies are required to elucidate their function in virus entry. Do they directly internalize viral particles or do they act in concert or sequentially with unknown molecules to form an entry complex that coordinates virus endocytosis? Which internalization pathways are exploited by incoming viral particles following TIM and TAM ligation? Are flaviviruses still internalized through clathrin-mediated endocytosis or targeted to alternative entry routes, such as macropinocytosis and phagocytosis? It will also be important to gain insight into the role of TAM-mediated signaling in the flavivirus infection of primary cells relevant for infection or, ideally, in animal models. What are the intracellular signaling cascades activated by TAM during virus entry? Are TAM receptors directly activated by flavivirus particles to shut down the host immune responses and facilitate viral spread? Finally, it is important to keep in mind that the expression of TIM and TAM receptors, as well as CLR cannot account for the tropism of flaviviruses, as several cellular models devoid of these molecules are permissive to flavivirus infection. This suggests that other receptors exist and remain to be discovered.
